# Gadoxetic Acid Disodium-Enhanced Magnetic Resonance Imaging for the Detection of Hepatocellular Carcinoma: A Meta-Analysis

**DOI:** 10.1371/journal.pone.0070896

**Published:** 2013-08-15

**Authors:** Xijiao Liu, Li Zou, Fei Liu, Yin Zhou, Bin Song

**Affiliations:** 1 Department of Radiology, West China Hospital, Sichuan University, Chengdu, Sichuan province, China; 2 Department of Orthopedics, West China Hospital, Sichuan University, Chengdu, Sichuan province, China; 3 Department of Liver and Vascular Surgery, West China Hospital, Sichuan University, Chengdu, Sichuan province, China; Yonsei University College of Medicine, Republic of Korea

## Abstract

**Objective:**

To determine the accuracy of MR imaging with gadoxetic acid disodium (Gd-EOB-DTPA) for the detection of hepatocelluar carcinoma (HCC).

**Materials and Methods:**

A systematic search was performed in PUBMED, EMBASE, Web of Science, Cochrane Library and the Chinese Biomedical Literature Database up to March 2013 to identify studies about evaluation of Gd-EOB-DTPA enhanced MR imaging in patients suspected of having HCC. The data were extracted to perform heterogeneity test and threshold effect test and to calculate sensitivity, specificity, diagnostic odds ratio, predictive value, and areas under summary receiver operating characteristic curve (AUC).

**Results:**

From 601 citations, 10 were included in the meta-analysis. The methodological quality of the 10 studies was good. **Overall HCC**: There was significant heterogeneity in the pooled analysis (I^2^ = 69.4%, P = 0.0005), and the pooled weighted values were determined to be sensitivity: 0.91 (95% confidence interval (CI): 0.89, 0. 93); specificity: 0.95 (95% CI: 0.94, 0.96); diagnostic odds ratio: 169.94 (95% CI: 108.84, 265.36); positive likelihood ratio: 15.75 (95% CI: 7.45, 33.31); negative likelihood ratio: 0.10 (95% CI: 0.06, 0.15). The AUC was 0.9778. **HCC in cirrhosis:** The estimates were to be sensitivity: 0.91 (95% CI: 0.88, 0.93); specificity: 0.93 (95% CI: 0.89, 0.95); diagnostic odds ratio: 234.24 (95% CI: 33.47, 1639.25); positive likelihood ratio: 15.08 (95% CI: 2.20, 103.40); negative likelihood ratio: 0.08 (95% CI: 0.03, 0.21). The AUC was 0.9814. **≤20** **mm HCC:** The AUC was 0.9936. There was no notable publication bias.

**Conclusions:**

This meta-analysis suggests that MR imaging with Gd-EOB-DTPA has high diagnostic accuracy for the detection of HCC, especially for ≤20 mm HCC. This technique shows good prospect in diagnosis of HCC.

## Introduction

Hepatocellular carcinoma (HCC) is the 5^th^ most common cancer and 3^rd^ most common cause of cancer death worldwide [1], [2]. Cirrhosis is the strongest predisposing factor for HCC, with approximately 80% of HCC developed in a cirrhotic liver [3]. The annual incidence of HCC is 2.0%–6.6% in patients with cirrhosis compared with 0.4% in patients without cirrhosis [3].

The importance of early detection of HCC has been emphasized [4], [5]. In practice, however, this can be challenging due to the high prevalence of benign lesions in cirrhotic livers and the variability of imaging features in HCC depending on their differentiation [6]. Magnetic resonance (MR) imaging, particularly contrast material-enhanced dynamic MR imaging, plays a crucial role in the accurate diagnosis of HCC [7]. A newly developed liver-specific hepatobiliary contrast agent, gadoxetic acid disodium (Gd-EOB-DTPA) (Primovist, Bayer Schering, Germany), is now available for use in hepatic MR examinations. Gd-EOB-DTPA is a gadolinium-based paramagnetic contrast agent that combines the properties of a conventional extracellular fluid contrast agent, thus enabling dynamic perfusion imaging, and a hepatobiliary agent, allowing evaluation of delayed hepatocyte uptake and biliary excretion [8]. It enters the hepatocytes through the organic anion transporting polypeptides OATP1B1 and OATP1B3, and excretes into the bile via the multidrug resistance protein 2 [9].

Previous studies showed Gd-EOB-DTPA-enhanced MR imaging had high diagnostic sensitivity and specificity for HCC [10], [11], [12], [13], [14], [15], [16], [17], [18], [19]. Meanwhile, there were studies suggesting Gd-EOB-DTPA-enhanced MR imaging had the same diagnostic performance as other contrast material-enhanced MR imaging [20], [21]. We designed a meta-analysis to evaluate the published experimental data regarding MR imaging with the use of Gd-EOB-DTPA for the detection of HCC in patients to determine diagnostic value of this imaging method and provide evidence of evidence-based medicine for clinical diagnosis.

## Materials and Methods

This meta-analysis was completed in accordance with the recommendations outlined in the Preferred Reporting Items for Systematic Reviews and Meta-Analyses statement [22].

### Literature Search Strategy

PUBMED, EMBASE, Web of Science, Cochrane Library and the Chinese Biomedical Literature Database were searched independently by two investigators (Xijiao Liu and Li Zou) using the terms ‘‘Gadolinium-EOB-DTPA OR gadoxetic acid disodium OR Gd-EOB-DTPA OR eovist OR primovist’’ and ‘‘Liver tumor OR hepatic tumor OR liver cancer OR hepatic cancer OR hepatocelluar carcinoma’’ (last search update March 13, 2013). The search involved the use of free-text words and MESH (Medical Subject Headings) terms for increased sensitivity of the search strategy. The search was without restriction to the language and on studies conducted on human subjects. Review articles, abstracts, case reports, letters, comments and unpublished articles were excluded. Extensive crosschecking of the reference lists of all retrieved articles was performed.

### Inclusion and Exclusion Criteria

Studies were included if, in addition, all of the following inclusion criteria were met: (a) Gd-EOB-DTPA-enhanced MR imaging with hepatobiliary phase (HBP) was performed to identify and characterize liver tumors; (b) histopathologic analysis (surgery, biopsy), and/or follow-up ultrasound, computed tomography (CT) or MR imaging was the reference standard; (c) the data were sufficient for the calculation of true-positive (TP), false-positive (FP), false-negative (FN) and true-negative (TN) values. Authors of studies with insufficient published data were contacted personally in an effort to retrieve the missing data. Studies were excluded if (a) any one of the inclusion criteria was not met; (b) multiple reports were published for the same study population (in this case, the publication with the most details and/or most recently published was chosen); and (C) the study included patients who had previously undergone treatment for liver tumors.

### Quality Assessment and Data Extraction

The methodological quality of the included studies was assessed independently by the same two investigators using Quality Assessment of Diagnostic Accuracy Studies (QUADAS) tool [23], [24]. Meanwhile, the relevant data were also extracted from each study, including: author, publication year, study nation, study population, study design type, magnetic field strength, type of coil used, pulse sequences, dose of Gd-EOB-DTPA, time for HBP, and descriptions of interpretations of the diagnostic tests. Disagreements were resolved by discussion between the two investigators.

For each study, values for TP, FP, FN, TN, sensitivity (SEN), specificity (SPE), positive likelihood ratio (PLR) and negative likelihood ratio (NLR) results for the detection of lesions were extracted, and 2×2 contingency tables were constructed.

### Statistical Analysis

First, we assessed the threshold effect, which arose when different cut-offs or thresholds were used in different studies to define a positive (or negative) test result. The Spearman correlation coefficient between the logit of SEN and the logit of (1- SPE) was computed to assess the threshold effect using Meta-Disc version 1.4 (http://www.hrc.es/investigacion/metadisc_en.htm). A strong positive correlation would suggest a threshold effect with P<0.05 [25], [26]. We constructed summary receiver operating characteristic (ROC) curve to assess SEN and (1- SPE) using the same software. The area under the ROC curve (AUC) was used to analyze the diagnostic precision of MR imaging with Gd-EOB-DTPA for the detection of HCC.

Exploring heterogeneity is a critical issue to understand the possible factors influencing accuracy estimates and to evaluate the appropriateness of statistical pooling of accuracy estimates from various studies. The Q statistic of the Chi-square value test and the inconsistency index (I-squared, I^2^) were used to estimate the heterogeneity of the individual studies by using Meta-Disc version 1.4. P<0.1 or I^2^>50% suggested notable heterogeneity [27]. If there were notable heterogeneities, the test performance was summarized by using a random-effects coefficient binary regression model; otherwise, a fixed-effects coefficient binary regression model was used [28]. The estimates for overall HCC, HCC in cirrhosis and ≤20 mm HCC were evaluated. Sensitivity analysis was performed in studies with a dose of 0.025 mmol Gd-EOB-DTPA per kilogram of body weight.

In this study, meta-regression analysis was used to determine characteristics that contributed to the heterogeneity. Co-variates (such as, study design, MRI field strength, dose of Gd-EOB-DTPA, enrollment patients) were used in the meta-regression.

The presence of publication bias was visually assessed by producing a Deeks funnel plot and an asymmetry test with the Stata software. Publication bias was considered to be present if there was a nonzero slope coefficient (P<0.05) [29].

## Results

### Characteristics of Studies

**Figure 1:**
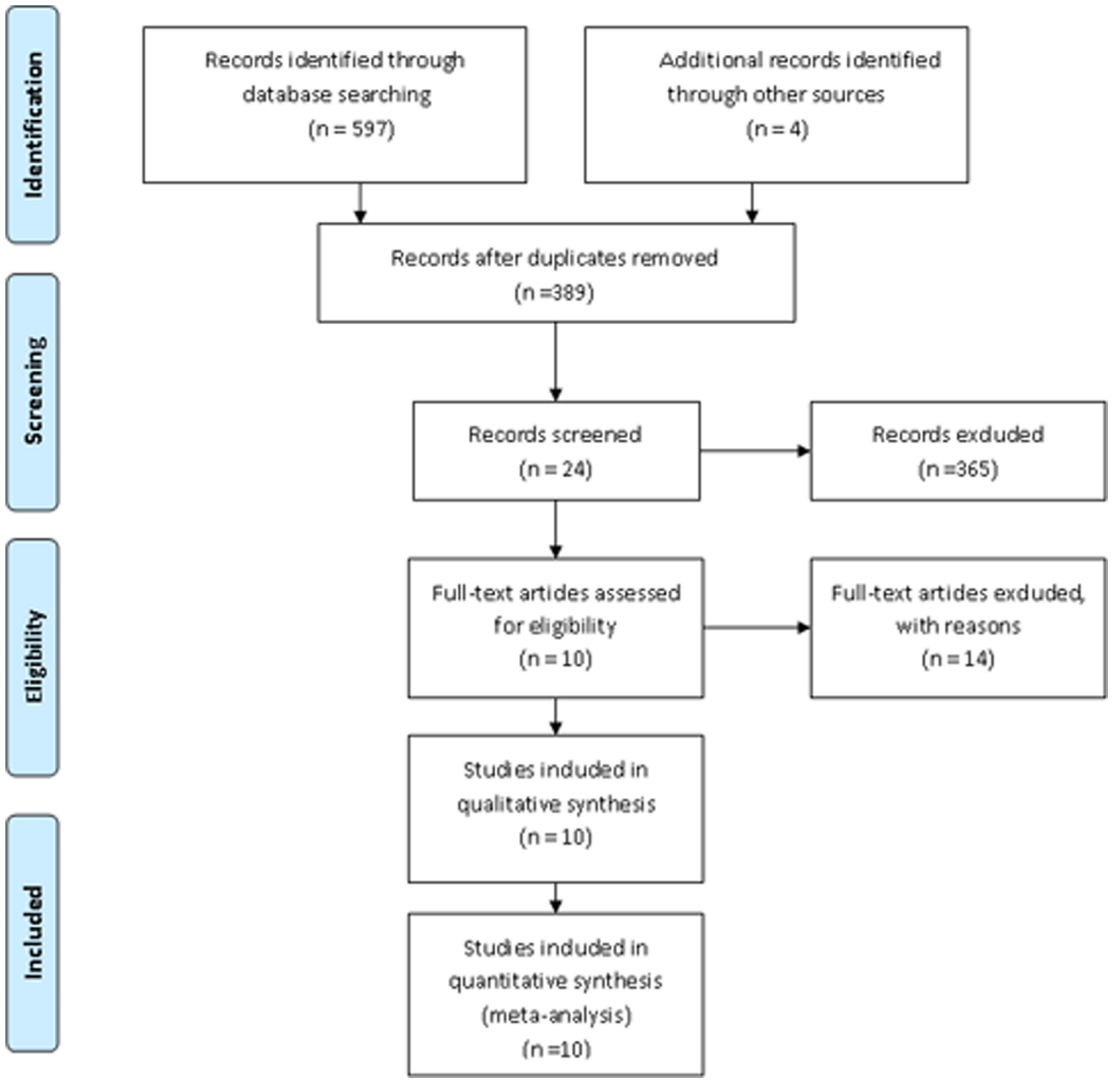
Flowchart illustrating the selection of studies.

**Figure 2:**
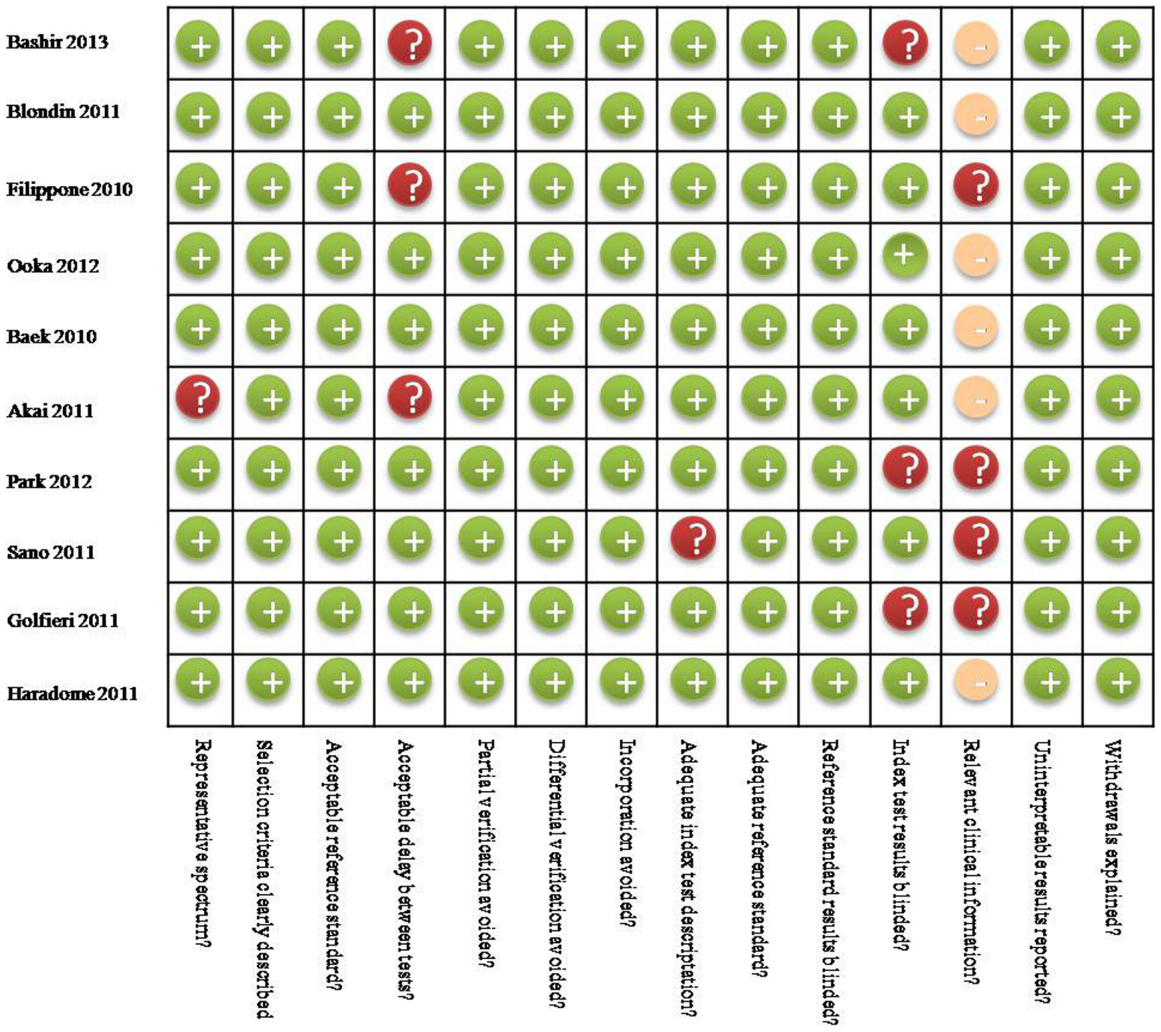
Methodological quality of the 10 included studies.

The systematic search that we conducted are summarized in Fig. 1. The search initially yielded 597 potential literature citations through database searching and 4 additional records was identified through grey literature searching. Two hundred and twelve studies were excluded for duplicates. After review of the titles and abstracts, 365 of these studies were excluded because they were not relevant studies. After reading the full texts, 14 of the remaining 24 articles were excluded since lacking sufficient information to complete a 2×2 contingency table. Finally, 10 studies were included in this study. The abstracted data of these individual studies are summarized in Table 1. All 10 primary studies fulfilled 11 to 13 items of the 14-item QUADAS checklist (Fig. 2).

**Table 1:** Characteristics of the included 10 studies.

No	Study	Year	Nation	TP	FP	FN	TN	No. of Tumors	Study Design	Magnetic field strength	Dose of Gd-EOB-DTPA	Patients		
												Enrollment	Ratio of Men to Women	Child-Pugh classification (A/B/C)
1	Bashir [9]	2013	American	64	14	6	41	125	retro	1.5/3.0T	10ml	consecutive cirrhosis patients,22mo (9/08–6/10)	57/43	ND
2	Blondin [11]	2011	Germany	37	1	4	5	47	retro	1.5T	10ml	consecutive patients,26mo (1/07–3/09)	25/8	22/9/2
3	Filippone [12]	2010	Italy	36	1	3	14	54	retro	1.5T	0.025mmol/Kg	cirrhosis patients,31mo (9/07–4/10)	27/7	21/8/5
4	Ooka [12]	2012	Japan	82	10	5	344	441	retro	1.5T	0.025mmol/Kg	consecutive patients,16mo (2/08–6/09)	40/14	46/8/0
5	Baek [13]	2010	Korea	63	4	10	55	132	retro	3.0T	0.025mmol/Kg	consecutive patients,13mo(5/08–6/09)	43/8	42/7/2
6	Akai [14]	2011	Japan	46	2	6	52	106	pro	1.5T	0.025mmol/Kg	consecutive patients,8mo (6/08–2/09)	27/7	ND
7	Park [15]	2012	Korea	147	2	32	142	323	retro	3.0T	0.025mmol/Kg	consecutive cirrhosis patients,13mo (5/09–6/10)	185/75	230/28/2
8	Sano [16]	2011	Japan	88	6	3	155	252	retro	1.5T	0.025mmol/Kg	consecutive patients,22mo (1/08–11/09)	47/17	54/10/0
9	Golfieri [17]	2011	Italy	172	2	1	40	215	pro	1.5T	0.025mmol/Kg	consecutive cirrhosis patients,13mo (5/08–10/09)	127/0	ND
10	Haradome [19]	2011	Japan	52	3	8	36	99	retro	1.5T	0.025mmol/Kg	consecutive patients,17mo (1/08–6/09)	60/15	48/5/22

TP, true-positive; FP, false-positive; TN, true-negative; FN, false-negative; ND, no data.

### Quantitative Synthesis

#### Overall HCC

**Figure 3:**
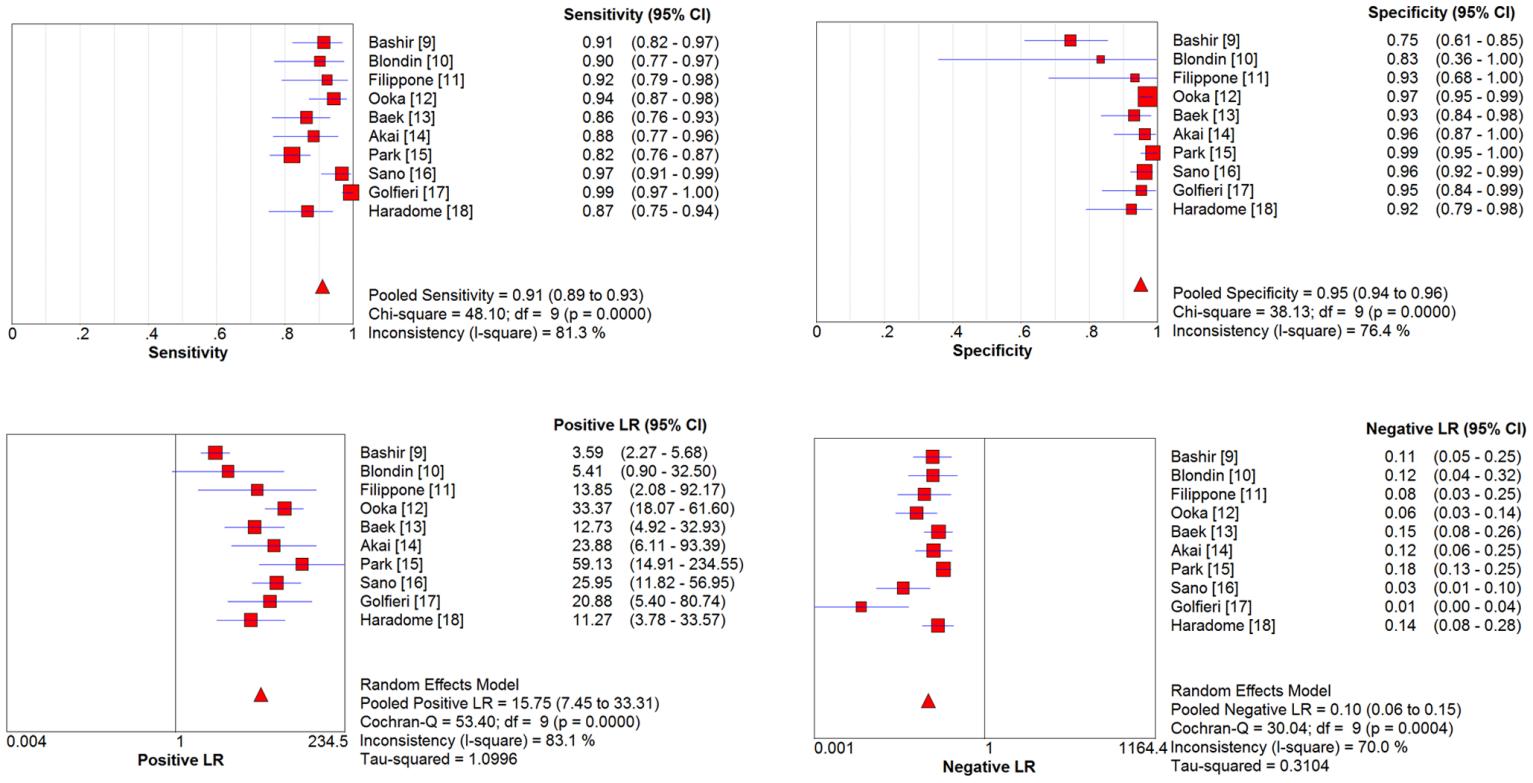
Forest plots of the estimates for MR imaging with Gd-EOB-DTPA for the detection of HCC.

**Figure 4:**
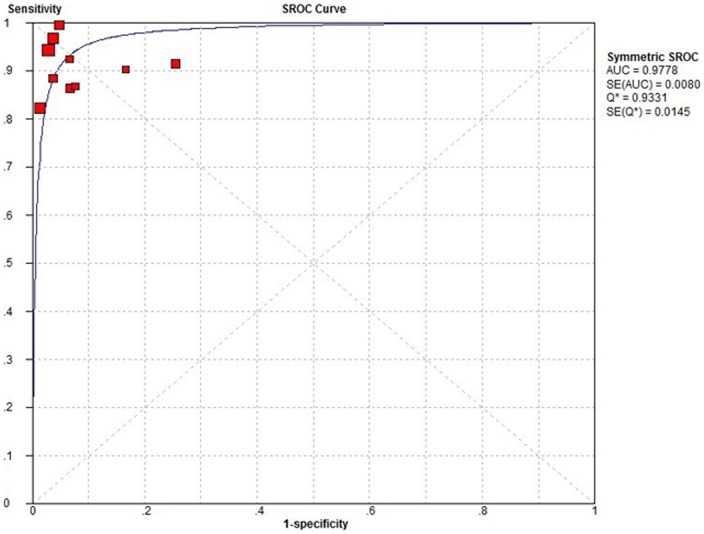
Summary receiver operating characteristic (SROC) curves of MR imaging with Gd-EOB-DTPA in detection of HCC.

A Spearman rank correlation was performed as a test for the threshold effect and was determined to be 0.03 (P = 0.934), which indicated that there was an absence of a notable threshold effect in the accuracy estimates among individual studies. Since there was significant heterogeneity in the pooled analysis for overall HCC (I^2^ = 69.4%, P = 0.0005), SEN, SPE, PLR, NLR were pooled by using a random-effects coefficient binary regression model in the 10 studies. The pooled weighted values were determined to be SEN: 0.91 (95% confidence interval (CI): 0.89, 0. 93); SPE: 0.95 (95% CI: 0.94, 0.96); DOR: 169.94 (95% CI: 108.84, 265.36); PLR: 15.75 (95% CI: 7.45, 33.31); NLR: 0.10 (95% CI: 0.06, 0.15). The forest plots from 10 studies are shown in Fig. 3. SROC curves are shown in Fig. 4. The AUC was 0.9778.

#### HCC in Cirrhosis

There were 4 studies [10], [12], [16], [18] which had been performed to focus on the accuracy of Gd-EOB-DTPA enhanced MR imaging for the detection of HCC in cirrhosis. Altogether, a total of 521 patients with 717 tumors were enrolled in this analysis. There was no notable threshold effect among individual studies (P = 0.800). Since there was significant heterogeneity in the pooled analysis (I^2^ = 81.3%, P = 0.0011), SEN, SPE, PLR, NLR were pooled by using a random-effects coefficient binary regression model. The estimates were to be: SEN, 0.91 (95% CI: 0.88, 0.93); SPE: 0.93 (95% CI: 0.89, 0.95); DOR: 234.24 (95% CI: 33.47, 1639.25); PLR: 15.08 (95% CI: 2.20, 103.40); NLR: 0.08 (95% CI: 0.03, 0.21). The AUC was 0.9814.

#### ≤20mm HCC

Only 3 studies [16], [17], [18] including 451 patients focused on the accuracy of Gd-EOB-DTPA enhanced MR imaging for the detection of ≤20 mm HCC. All nodules were confirmed pathologically. There was significant threshold effect in the accuracy estimates among 3 studies (P<0.05). We only constructed SROC curve to assess SEN and (1- SPE). The AUC was 0.9936.

### Sensitivity Analysis

Among the included studies, two [10], [11] used a fixed dose of 10 mL of Gd-EOB-DTPA. While in the other 8 studies, Gd-EOB-DTPA was administrated according to the manufacturer`s instructions for a dose of 0.025 mmol per kilogram of body weight. Here we conducted sensitivity analysis for the 8 studies.

There was an absence of a notable threshold effect in the accuracy estimates among 8 studies (P = 0.955). Since significant heterogeneity existed in the pooled analysis for overall HCC (I^2^ = 52.7%, P = 0.0387), SEN, SPE, PLR, NLR were pooled by using a random-effects coefficient binary regression model. The pooled weighted values were determined to be SEN: 0.91 (95% CI: 0.89, 0. 93); SPE: 0.97 (95% CI: 0.95, 0.98); DOR: 285.32 (95% CI: 130.48, 623.88); PLR: 23.73 (95% CI: 16.71, 33.72); NLR: 0.09 (95% CI: 0.05, 0.15). The AUC was 0.9871.

### Heterogeneity Analysis and Publication Bias

There was significant heterogeneity in the pooled analysis for overall HCC. The results of meta-regression analysis showed that study design, MRI field strength, tumor diameter or patients did not contribute to the heterogeneity statistically (P>0.05). However, dose of Gd-EOB-DTPA contributed significantly to the heterogeneity (P = 0.0266).

**Figure 5:**
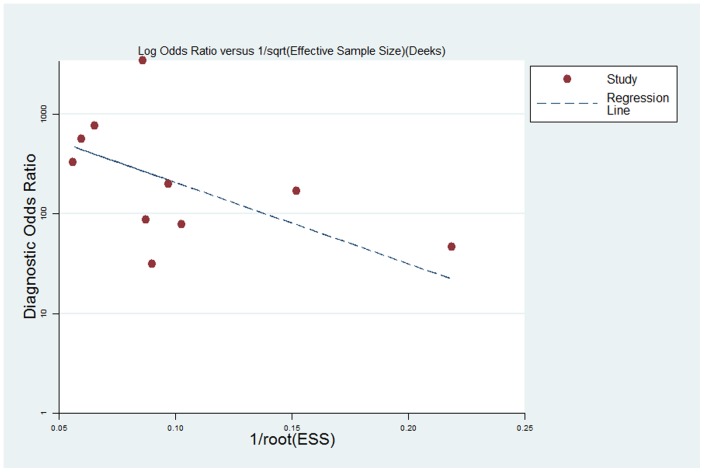
The funnel plot of publication bias: Linear regression of the inverse root of effective sample sizes (ESS) on a log DOR is performed.

The results of Deeks funnel plot asymmetry test (P = 0.237) showed evidence of no notable publication bias (Fig. 5).

## Discussion

To the best of our knowledge, this is the first meta-analysis of the diagnostic performance of MR imaging with Gd-EOB-DTPA to assess HCC. Results of our study showed that MR imaging with Gd-EOB-DTPA had high pooled sensitivity (0.91 (95% CI: 0.89, 0. 93)) and high pooled specificity (0.95 (95% CI: 0.94, 0.96)) for the detection of HCC, especially for ≤20 mm HCC. The included studies had high quality. The studied patients were mostly consecutively enrolled from general hospital populations and were thus representative of clinical practice in a hospital setting.

There was significant heterogeneity in the pooled analysis of the included 10 studies. Spearman correlation coefficient demonstrated no significant threshold effect exists. Meta-regression analysis showed that dose of Gd-EOB-DTPA contributed significantly to the heterogeneity. Two of the studies [10], [11] used a fixed dose of 10 mL of Gd-EOB-DTPA. While in the other 8 studies, Gd-EOB-DTPA was administrated according to the manufacturer`s instructions for a dose of 0.025 mmol per kilogram of body weight. The results of sensitivity analysis for the 8 studies were similar with the results for the 10 studies, which suggested our study results were reliable.

Among the included studies in our meta-analysis, Golfieri *et al*. [18] reported the highest diagnostic odds ratio. We speculated this result might correspond to the gender composition of enrollment patients. All participants in the study were men with mostly HBV/HCV-related cirrhosis. Epidemiology statistics show liver cancer is more prevalent in male than female [2], [30]and infection with the hepatitis B and C viruses is the major risk factors, which increase the risk of liver cancer some 20-fold [31].

Sugimoto *et al*. [32], [33] reported both contrast-enhanced ultrasound and Gd-EOB-DTPA-enhanced MR imaging had comparable abilities in the characterization of non-hypervascular HCC. Nevertheless, contrast-enhanced ultrasound yielded a significantly higher diagnostic accuracy in the assessment of arterial hypervascularity of lesions. CT and MR imaging are the modalities of choice for the diagnosis and follow-up of patients with HCC. Studies found Gd-EOB-DTPA-enhanced MR imaging had improved diagnostic accuracy compared with CT for the detection of HCC, particularly for smaller lesions [19], [34], [35]. Gd-EOB-DTPA-enhanced MR imaging yielded fewer false-positive findings than CT, although there was no statistically significant difference in positive predictive value. Along with improved capabilities in the detection of HCC, an additional benefit of MR imaging compared with CT is the absence of radiation hazards.

In 1996, Thomas *et al*. [36] published a prospective, double blinded, randomized trial study to compare the usefulness of Gd-EOB-DTPA and gadopentetic acid (Gd-DTPA) in the diagnosis of focal liver lesions in 31 patients and found Gd-EOB-DTPA-enhanced MR imaging enabled improved detection of hepatic lesions over that of Gd-DTPA while providing comparable differential diagnostic information. In 2002 [37] and 2010 [38], similar findings were reported. In 2010, Kim *et al*. [39]reported that the area under ROC curve (Az value) and sensitivity of Gd-EOB-DTPA-enhanced MR imaging for the detection of ≤3 cm HCCs were 0.964 and 90.7%, which were significantly higher than those of the SPIO-enhanced MR imaging (Az 0.830; sensitivity 84.7%). In 2010, Antonella *et al*. [40] published a multicenter randomized trial to compare the efficacy of Gd-EOB-DTPA and gadobenate dimeglumine in contrast enhanced MR imaging of the liver. They found that in the HBP, liver enhancement after injection of Gd-EOB-DTPA was superior to that obtained with gadobenate dimeglumine. In summary, above reports support superior diagnostic performance of Gd-EOB-DTPA-enhanced MR imaging compared to other contrast material enhanced MR imaging and HBP images provided modest improvement in the diagnosis of HCC [18], [41].

Although MR imaging with Gd-EOB-DTPA has high diagnostic accuracy for the detection of HCC, it is not perfect. Park *et al*. [16] retrospectively analyzed gadoxetic acid-enhanced MR imaging of 179 surgically confirmed small HCCs in cirrhosis, 11 HCCs were not verified by observers. Reviews of these lesions found that 5 of them were not seen on images and remaining 6 were seen as subtle arterially enhanced nodules or as hypointense only on HBP images. Eight of these HCCs were histological confirmed at liver transplantation in 6 patients classified as having Child-Pugh class B or C cirrhosis. Gd-EOB-DTPA uptake in liver parenchyma is significantly associated with liver function and liver enhancement is lower in Child-Pugh class C group [42], [43], [44], [45], [46]. Hence, the contrast signal between tumor and parenchyma in HBP in severe liver dysfunction patients is reduced, leading to overall diagnostic accuracy of Gd-EOB-DTPA-enhanced imaging of focal liver lesions depressed in these patients. Therefore, if the result of the index test is negative but a high level of clinical suspicion of HCC remains in patients with severe liver dysfunction, the negative index test result should not prevent other imaging modalities such as dynamic CT or ultrasound.

With currently available imaging criteria for HCC such as the Association for the Study of Liver Diseases criteria [47], [48], the diagnosis of malignancy applies only for nodules that are larger than 10 mm and show typical vascular profiles. Therefore, diagnoses of ≤10 mm HCCs are still made on the basis of positive biopsy results or patients with those lesions are recommended for follow-up examination. This emphasizes the need to refine new diagnostic parameters for ≤10 mm HCC. Two of the included studies in this meta-analysis reported a sensitivity of 100% with a specificity of 100% [18] and a sensitivity of 61.8% with a specificity of 96.7% [16] for detection of ≤10 mm HCC using Gd-EOB-DTPA-enhanced MR imaging. In summary, the reported diagnostic accuracy of ≤10 mm HCC by MR imaging with Gd-EOB-DTPA is quite heterogeneous [16], [18], [49]. Further researches with large sample size are needed to verify this application.

Some limitations of this meta-analysis should be addressed. First, the moderate sample size. However, the study quality of the included studies was generally high. Moreover, a discussion of a systematic review that studied the characteristics of meta-analyses and their included studies in the Cochrane Database showed that the number of studies eligible for meta-analysis is typically very small in all medical areas. Second, participants in the included studies were suspected of having HCCs on the basis of ultrasound, CT or alpha-fetoprotein findings obtained during routine HCC workup, which might have caused selection bias. Third, limited numbers of lesions were diagnosed at liver transplantation, which might have resulted in overestimation of the diagnostic performance of MR imaging by decreasing the number of false-negative lesions. Finally, as per the manufacturer`s labeling instructions, all the HBP imaging for included five studies was performed 20 minutes after contrast medium administration. Although this interval has been shown to yield the highest conspicuity for liver tumors in patients with normal liver function [50], recent evidence indicates that contrast medium uptake and biliary excretion may be delayed in patients with cirrhosis.

In conclusion, our meta-analysis showed that MR imaging with Gd-EOB-DTPA had high diagnostic accuracy for the detection of HCC, especially for ≤20 mm HCC. Even though the number of the included studies is small, MR imaging with Gd-EOB-DTPA shows good prospect in diagnosis of HCC.

## Supporting Information

Checklist S1 **PRISMA 2009 Checklist.**
(DOC)
